# Investigations of Sidewall Passivation Using the Sol-Gel Method on the Optoelectronic Performance for Blue InGaN Micro-LEDs

**DOI:** 10.3390/mi14030566

**Published:** 2023-02-27

**Authors:** Wenjun Huang, Xiangyu Miao, Zhaojun Liu

**Affiliations:** Department of Electronic and Electrical Engineering, Southern University of Science and Technology, Shenzhen 518055, China

**Keywords:** sidewall passivation, sol-gel SiO_2_, GaN-based micro-light-emitting diodes (Micro-LEDs)

## Abstract

The optoelectronic effects of sidewall passivation on micro-light-emitting diodes (Micro-LEDs) were investigated using sol-gel chemical synthesis. Blue InGaN/GaN multi-quantum well (MQW) Micro-LEDs, ranging in size from 20 × 20 μm to 100 × 100 μm and with high EQE, were fabricated and distinguished by the passivation method used, including no passivation, sol-gel SiO_2_, and plasma-enhanced chemical vapor deposition (PECVD) SiO_2_. Impressively, the sol-gel method is advantageous in improving the optoelectronic performance of Micro-LEDs. The fabricated 20 × 20 μm Micro-LEDs showed an EQE of 27.7% with sol-gel passivation, which was a 14% improvement compared to devices without sidewall passivation. Sol-gel sidewall passivation allows Micro-LEDs to effectively achieve sharper edge emission, superior surface luminous uniformity, and intensity, providing the possibility for the fabrication of low-cost and high-efficiency Micro-LEDs.

## 1. Introduction

In recent years, GaN-based micro-light-emitting diodes (Micro-LED) displays have emerged as a promising platform of next-generation displays due to their superior optical and physical properties such as low power consumption, high luminous efficiency, and fast response [1,2,3]. These features make Micro-LEDs a compelling technology for high-resolution near-eye displays for augmented and virtual reality (AR/VR) and visible-light communication [4,5,6]. To achieve the high resolution and immersive demands for human–machine interface, individual pixels need to be shrunk down to micrometer sizes while maintaining exceptional efficiency and brightness [7].

However, small-sized Micro-LEDs with a size of less than 100 µm suffer from decreased external quantum efficiency (EQE) due to increased surface-to-volume ratio, leading to increased Shockley–Read–Hall (SRH) non-radiative recombination at the surface of the active layer. This problem is mainly caused by sidewall damage, which is known to occur during fabrication through plasma-assisted etching, resulting in non-radiative recombination centers, such as defects, impurities, and dangling bonds [8,9,10,11,12,13,14]. Therefore, it is of great significance to minimize those sidewall defects that occur during fabrication and protect the surface with a stable passivation layer. A variety of studies have been devoted to reducing the sidewall damage of Micro-LEDs and dramatic improvements in the EQE have been demonstrated [15,16,17]. For example, Wong et al. used atomic-layer deposition (ALD) for sidewall passivation, after which devices’ EQE increased from 24% to 33% for 20 × 20 µm [18]. Wet chemical treatment such as potassium hydroxide (KOH) or TMAH solution, also used for reducing the sidewall damage induced from the inductively coupled plasma (ICP) etching, can achieve less than 10% improvement for EQE [19,20]. However, as the size decreased to less than 10 µm, EQE sharply dropped to less than 20% [21]. Especially down to 1 µm, EQE was less than 3% [22]. Recently, the sol-gel method has been used to passivate the sidewall of GaN-LEDs, resulting in a high EQE and improved performance. This method has advantages over ALD and plasma-enhanced chemical vapor deposition (PECVD) as it can be carried out at room temperature and has many advantages in defect optimization. For instance, Sheen et al. demonstrated a blue nanorod-LED array with a high EQE of 20.2%, the highest value ever reported for the LED in the nanoscale, using the sol-gel method [23]. This sol-gel method is advantageous for sidewall passivation on GaN-LEDs because SiO_2_ nanoparticles are absorbed on the surface, which minimizes atomic interaction with the GaN surface and passivates dangling bonds. Thereby, high-performance LEDs were manufactured with minimized surface defects and decreased non-radiative recombination centers. In addition to these methods, other techniques, such as the use of a tunnel junction and ion implantation, have been proposed to mitigate sidewall damage and enhance device performance. Tunnel junctions have been shown to reduce the resistive loss of the device and increase the efficiency of Micro-LEDs [24]. Ion implantation has also been explored as a means of confining injected current and mitigating sidewall damage [25]. Despite the significant progress made in addressing the issue of sidewall damage in Micro-LEDs, continued research is necessary to further improve the performance and reliability of these devices.

In this paper, we investigate the effects of sidewall passivation on Micro-LEDs by the sol-gel method and observe the optoelectronic and efficiency characteristics while studying the size-dependent effects. Our study involves designing Micro-LEDs with sol-gel passivation, PECVD passivation, and without passivation, with sizes ranging from 20 × 20 µm to 100 × 100 µm. We show that for 20 × 20 µm Micro-LEDs, the peak EQE and wall-plug efficiency (WPE) were 27.7% and 27.1%, respectively, with sol-gel passivation, compared to 13.4% and 10.3% without sidewall passivation. Our results show that the sol-gel method minimizes surface defects and reduces non-radiative recombination centers, which has implications for future research on the use of the sol-gel method for Micro-LED sidewall passivation. Additionally, we also compared the surface emission uniformity of different passivation methods and observed that Micro-LEDs exhibit superior surface emission uniformity with sol-gel passivation.

## 2. Materials and Methods

**Figure 1:**
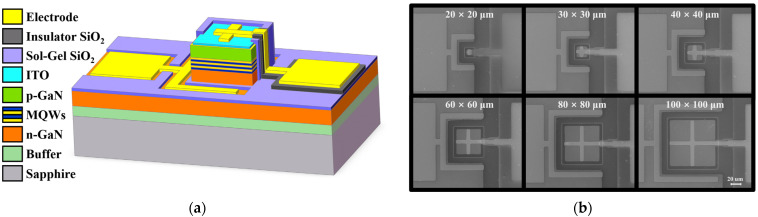
(**a**) Cross-section schematics of the Micro-LED design; (**b**) SEM images of Micro-LEDs with sol-gel SiO_2_ passivation.

**Figure A1:**
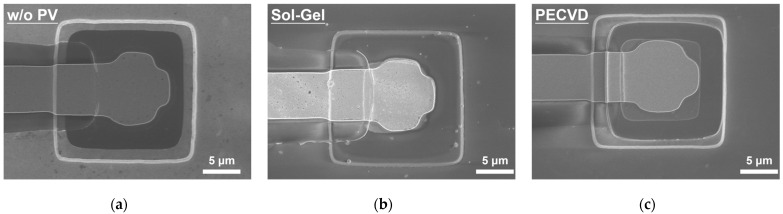
SEM images of Micro-LEDs (**a**) without passivation, (**b**) with sol-gel SiO_2_ passivation, (**c**) with PECVD SiO_2_ passivation.

In our study, Micro-LED structures were fabricated on commercial c-plane InGaN blue LED epitaxial wafer (Changelight Co., Ltd., Xiamen, China) grown on sapphire substrate. Micro-LEDs were fabricated with a size range from 20 × 20 µm to 100 × 100 µm. The details of device structures with sol-gel SiO_2_ passivation are shown in Figure 1a. Additionally, Figure 1b provides a top-view of the Micro-LEDs with sol-gel SiO_2_ passivation. These images demonstrate the uniformity and consistency of the passivation process. Moreover, we have also included SEM images of 20 × 20 µm Micro-LEDs with different passivation structures in Figure A1 of Appendix A.

Different sizes of micro patterns were constructed through lithography by Micro Writer ML3 and then it was dry-etched by ICP using SiO_2_ as a hard mask. After cleaning the mask with buffered HF (BOE), 110 nm indium-tin oxide (ITO) was deposited on the p-GaN via magnetron sputtering and annealed at 600 °C with 80% N_2_ and 20% O_2_ for 5 min using rapid thermal annealing to achieve ohmic contact. Then, ~100 nm SiO_2_ sidewall passivation was individually performed by sol-gel synthesis and PECVD. After exposing ITO and n-GaN via ICP etching, 320 nm Ti/Al/Ti/Au was deposited by electron beam (e-beam) deposition on ITO and n-GaN as p- and n-electrodes. In order to achieve good reliability and consistency of the device structure between with and without passivation layer, a 150 nm SiO_2_ insulating layer was deposited under the p-electrode before e-beam deposition. Here, three groups of samples distinguished by passivation layer were prepared, and each group with size range from 20 × 20 µm to 100 × 100 µm. Groups were labeled as S1/S2/S3, in which S1 stands for the device without passivation layer, and S2 and S3 stands for the device with passivation layer formed by PECVD and sol-gel method, respectively. Here, S1/S2/S3 were respectively fabricated from three small pieces cut from one same 4-inch epitaxial wafer.

For the preparation of SiO_2_ passivation layer, S2 was deposited by PECVD, using SiH_4_ and N_2_O as precursors reacted at 350 °C, and S3 was formed by sol-gel chemical synthesis method at room temperature. For the synthesis of sol-gel SiO_2_ passivation layer [18], tetraethyl orthosilicate (TEOS, Macklin, Shanghai, China 98%) was used as a precursor. First, 52.5 mL anhydrous ethanol (EtOH) and 72.5 mL deionized water were mixed in a beaker, and the sample immersed in the solution. Then, 0.2 g hexadecyl trimethyl ammonium bromide (CTAB, Macklin, 99%) was dissolved in the solution. After stirring for 10min at 1000 rpm until the CTAB was completely dissolved, 0.625 mL ammonia solution (Aladdin, Shanghai, China, 28–30%) and 0.325 mL TEOS were sequentially added dropwise into the solution and stirred at 1200 rpm at room temperature for 2 h. The final sol-gel SiO_2_ passivation (~100 nm) was obtained by twice repeating the above steps. Lastly, the samples were cleaned with dimethyl sulfoxide (DMSO), EtOH, and deionized water.

## 3. Results and Discussion

To distinguish the effect of passivation on the performance of Micro-LED devices, three different types of devices were fabricated: S1 is used for Micro-LEDs without passivation, S2 is labeled for Micro-LEDs with PECVD sidewall passivation, and S3 is used to designate the Micro-LEDs with sol-gel sidewall passivation. Using analysis methods such as electroluminescence (EL), current-voltage (I-V), external quantum efficiency (EQE), and wall plug efficiency (WPE) [10,26], we compared the device characteristics of Micro-LEDs with different sizes and passivation types, including without passivation, sol-gel SiO_2_, and PECVD SiO_2_. Our results showed that sol-gel passivation has considerable optimization potential for Micro-LEDs.

**Figure 2:**
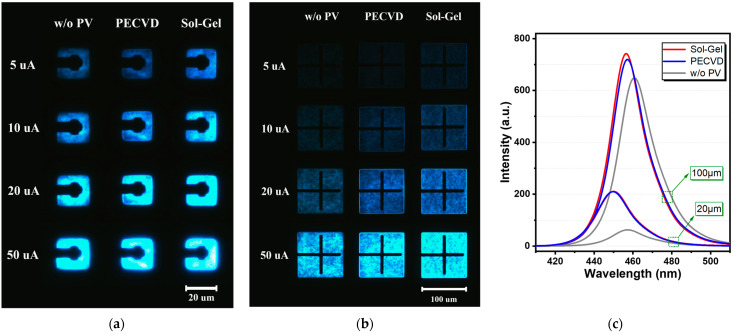
(**a**,**b**) EL emission images of S1, S2, and S3 for (**a**) 20 × 20 µm and (**b**) 100 × 100 µm; (**c**) EL spectrum of S1, S2, and S3 for 20 × 20 µm (at 50 A/cm^2^) and 100 × 100 µm (at 10 A/cm^2^).

**Figure A2:**
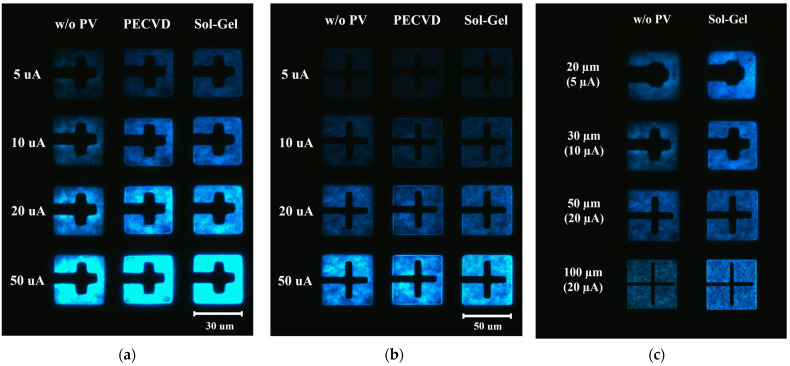
EL emission images of S1, S2, and S3 for (**a**) 30 ×30 µm, (**b**) 50 × 50 µm, and (**c**) low-current density emission.

**Figure A3:**
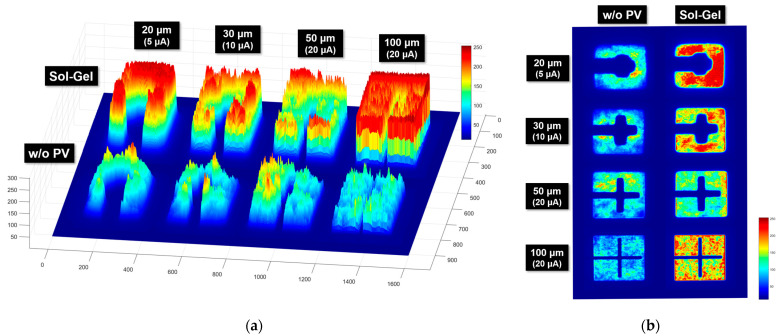
EL emission images of S1, and S3 presented as a 3D stereogram in (**a**) and a 2D intensity profile in (**b**).

Figure 2a,b show the EL composite images of S1, S2, and S3 for 20 × 20 µm and 100 × 100 µm Micro-LEDs illuminated at different currents ranging from 5 µA to 50 µA. Appendix A Figure A2 contains additional images to provide a more comprehensive understanding of the improved emission profile. The exposure time and other parameters were the same for all images to ensure that the different driving currents can be compared. To highlight the difference between passivated and non-passivated devices, the devices were also only taken at lower current density, as shown in Figure A2c. Emission characteristics of Micro-LEDs obtained from the top region confirm that the optical performance of the devices with passivation has been manifestly improved, which can be observed in S2 and S3, specifically in terms of surface luminous uniformity and emission intensity. Intensity optimization can be obviously observed from Figure A3a. In contrast, S1 shows a considerable brightness difference and poor uniformity between the central and border areas, especially at lower injection conditions. The border area also suffers from relatively dark emission, and there is no obvious edge interface feature observed, mainly due to the sidewall defects. With SiO_2_ sidewall passivation, the number of defects on the sidewall surface is reduced, resulting in a better edge interface emission effect that can be observed in S2 and S3. Although there is still some brightness difference between the central and border area in S2 like 20 × 20 µm at 20 µA, it is significantly reduced compared to S1. S3 shows a superior improvement in terms of the uniformity of light emission, and the luminous intensity is also significantly better in passivated devices. Smaller devices in S1, as seen in Appendix A Figure A2, display clear dark edge emission that can be improved by sol-gel passivation. However, in larger devices such as 100 × 100 µm, the effect of sidewall passivation on edge emission is not as pronounced, which is consistent with the efficiency improvement and EL intensity. Overall, sol-gel passivation improves the intensity in all size surface emission cases, compared to PECVD and non-passivated devices, and can improve the edge-to-center surface uniformity in smaller sizes.

Figure 2c shows the EL intensity of three types of devices, which were obtained using an integrating sphere, and we can observe that devices with sidewall passivation show a better EL intensity, confirming that the optical performance of the devices is improved by passivation. To ensure accurate and comparable intensity measurements, we set the testing current for the EL intensity at 50 A/cm^2^ (200 µA) for the 20 × 20 µm devices and 10 A/cm^2^ (1000 µA) for the 100 × 100 µm devices. This approach allowed us to overcome the sensitivity limitation of the equipment for non-passivated devices, which showed low emission intensity and required a higher current to obtain reliable and accurate data. It is worth noting that the improvement in EL intensity is more evident in the 20 × 20 µm devices, while the larger devices 100 × 100 µm show a slight improvement of passivation. It is commonly observed that smaller devices have a higher active-region-to-non-active-region ratio, leading to a higher current density and a more significant impact of sidewall defects on device performance. We believe that the difference in improvement between the 20 × 20 µm and 100 × 100 µm devices can potentially be explained by the dead zone ratio [21] and revised active region area ABC model [22]. These could explain why the smaller devices in our study exhibited a more pronounced enhancement in performance with sol-gel passivation than the larger devices. The effectiveness of sidewall passivation may also depend on the type and density of sidewall defects present in the device, which could vary between different sizes of Micro-LEDs. Further research is needed to better understand these phenomena.

**Figure 3:**
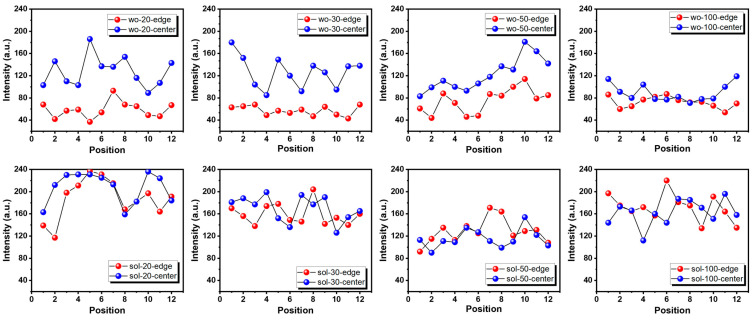
Intensity distribution of edge (red dot) and center (blue dot) emission of different sizes with different passivation.

**Figure A4:**
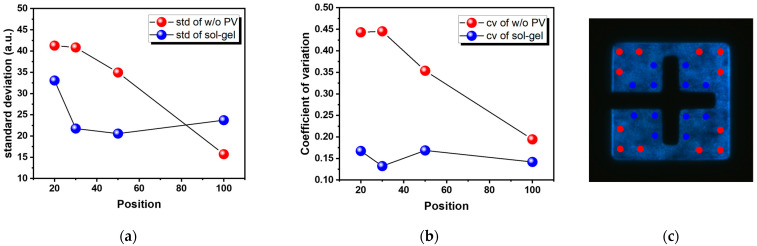
(**a**) Standard deviation and (**b**) coefficient of variation of Micro-LEDs with different passivation and sizes, (**c**) presents the results of our point selection method, which divides the edge (red) and center (blue) regions.

To assess the uniformity of the Micro-LEDs, we compared devices with and without sol-gel passivation by analyzing the obtained intensity information. The information was calculated using imaging analysis in MATLAB, as shown in Figure A3. We then compared the edge and center region emission intensity through statistical analysis and obtained the standard deviation (std) and coefficient of variation (cv), which describe the uniformity of the emission surface. From Figure 3, we can see that the emission uniformity was improved with sol-gel passivation, as the luminous intensity between the edge and the center was more coincident. For example, for 20 µm and 30 µm, the standard deviation for without passivation was 41.3 and 40.8, respectively, while for sol-gel passivation, they were 33.1 and 21.8, representing a significant optimization. We also calculated the coefficient of variation by dividing the standard deviation by the average intensity and found that it decreased from 44.3% and 44.5% for without passivation to 16.7% and 13.3% for sol-gel passivation, respectively, indicating better uniformity. More information about the point selection method, as well as the std and cv calculations, can be found in Figure A4. This improvement in surface uniformity is expected to have a positive impact on the overall performance and reliability of the Micro-LEDs.

**Figure 4:**
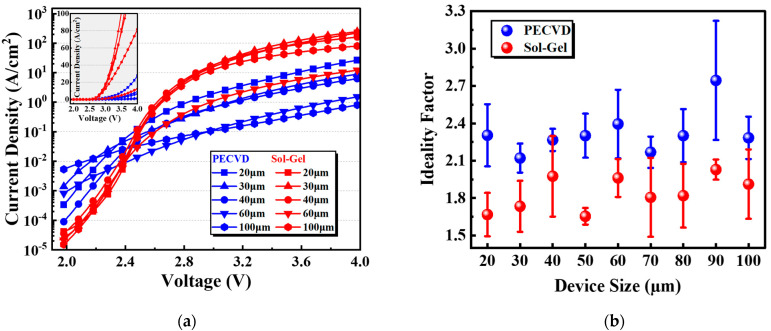
(**a**) Current density vs. voltage (J–V) characteristics of S2 and S3 for different sizes in log scale and linear scale; (**b**) ideality factor of S2, and S3 with different sizes.

Electrical characteristics of Micro-LEDs with sidewall passivation are presented in Figure 4. Micro-LEDs with sol-gel SiO_2_ show a lower leakage current than PECVD SiO_2_ at below-threshold voltages [27], as shown in Figure 4a. At this region, with the increase in forward voltage, the current density of S3 increases more rapidly than S2. This is consistent with the decrease in the ideality factor of Micro-LEDs with the sol-gel SiO_2_ of 1.67 compared with that of the PECVD SiO_2_ of 2.30, as is shown in Figure 4b. The ideality factors [28] were calculated from the forward current density–voltage characteristics of the Micro-LEDs using Equation (1):

(1) $$n=\frac{q}{kT}{\left(\frac{\partial \ln J}{\partial V}\right)}^{-1}$$

where *n* is the ideality factor, *q* is the elementary charge, *k* is the Boltzmann constant, *T* is the absolute temperature in Kelvin, *J* is the current density, and *V* is the voltage. The ideality factor is a useful indicator to determine the effectiveness of sidewall treatments. The decrease in the ideality factor of S3 indicates that SRH recombination declined, and carrier recombination was likely the dominant mechanism [29,30]. Thereby, S3 exhibited the superior luminous intensity and sharper edge emission in Figure 2a,b.

**Figure 5:**
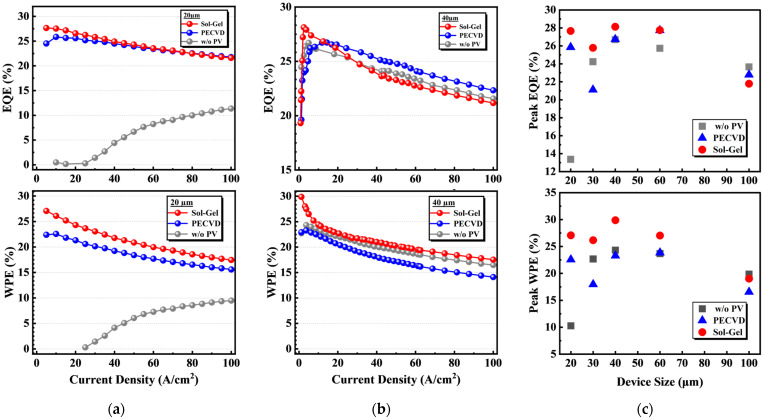
EQE and WPE curves of Micro-LEDs of S1, S2, and S3 for (**a**) 20 × 20 µm and (**b**) 40 × 40 µm; (**c**) peak EQE and WPE of S1, S2, and S3 for different sizes.

**Figure A5:**
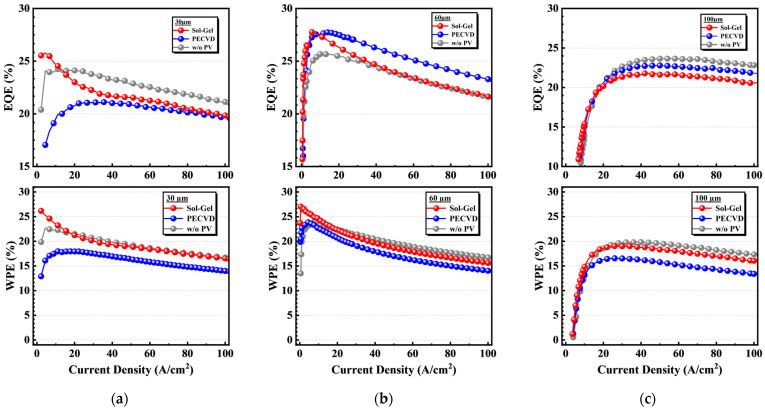
EQE and WPE curves of Micro-LEDs of S1, S2, and S3 for (**a**) 30 × 30 µm and (**b**) 60 × 60 µm; (**c**) 100 × 100 µm.

Figure 5 demonstrates the efficiency characteristics of Micro-LEDs with three passivation types. Figure 5a,b compare the EQE and WPE curves for Micro-LEDs with different surface passivation types whose sizes distribution are 20 × 20 µm and 40 × 40 µm, and Figure 5c shows the peak EQE and WPE of S1, S2, and S3 for different sizes. Figure A5 in Appendix A includes EQE and WPE data for Micro-LEDs with sizes other than those presented in the main text. The size of 20 × 20 µm from S2 and S3 shows a considerable improvement in peak EQE and WPE compared to S3. The peak EQE of S1, S2, and S3 are 27.7%, 25.8%, and 13.4% individually, which indicate that sol-gel SiO_2_ has the best optimization characteristic at around 10 A/cm^2^ and even higher, which have advantages for display applications at low current density [31]. The peak WPE of S1, S2, and S3 are 27.1%, 22.6%, and 10.3% individually. The effect of this efficiency improvement trend is more obvious in WPE curves. As the size became bigger, for 40 × 40 µm, S3 still achieves the highest peak EQE and WPE but S2 became more unstable and scattered. Even at lower current density, S2 shows a lower EQE than S3. As the plasma damage during the PECVD deposition process was still present after sidewall passivation, SRH non-radiative recombination through sidewall damage still contributed to the efficiency loss [18].

As the surface-to-volume ratio increases for smaller chip sizes, the influence of the sidewall damage on the device performance became more significant [22,32]. Therefore, smaller-size devices urgently need sidewall passivation to optimize and achieve higher device efficiency. As shown in Figure 5c peak EQE distribution, sol-gel SiO_2_ passivation can still obtain the highest peak EQE except for 100 × 100 µm. The Micro-LEDs without sidewall passivation showed a trend that the peak EQE decreases gradually for the devices smaller than 40 × 40 µm, while sol-gel SiO_2_ sidewall passivation had a distribution of the peak EQEs between 28.1% and 25.8% with relatively small variation [19]. The improvement for 20 × 20 µm can reach 14.3% for EQE and 16.8% for WPE, which is impressive for small-size Micro-LEDs.

Regarding the optimization of device performance, it is because the sol-gel process provides effective passivation for the GaN surface. During the passivation process, SiO_2_ particles are absorbed on the GaN surface, which can greatly reduce its atomic interaction and passivate the dangling bond hanging on the surface, thereby resulting in a low leakage current, decreased surface defects, and high EQE and WPE.

Although the sol-gel SiO_2_ passivation provides superior optoelectronic properties such as high brightness emission with high uniformity, high peak EQE, a low leakage current at below-threshold voltages, and lower ideality factor, it still has a few disadvantages. One of these is that the efficiency drops more severely at a higher current injection. This may be related to a thermal effect, which we plan to investigate further through imaging the devices with a thermal camera or by using a finite element analysis (FEA) thermal model to better understand this phenomenon. Additionally, while sol-gel passivation leads to improved performance for device sizes up to 30 × 30 µm, limited efficiency optimization for large sizes remains a challenge. Further studies are needed to obtain further improvement and optimize the efficiency of large devices through process optimization and defects analysis.

## 4. Conclusions

In summary, our study demonstrates that sol-gel sidewall passivation is a highly effective method for improving the efficiency and performance of Micro-LEDs. With sol-gel passivation, we observed a significant improvement in peak EQE and WPE for 20 × 20 µm Micro-LEDs compared to non-passivated devices, as well as sharper edge emission and superior surface luminous uniformity and intensity. In addition, this method helps to reduce current leakage and the ideality factor to 1.67. These results highlight the potential of sol-gel SiO_2_ sidewall passivation in reducing SRH non-radiative recombination and surface recombination induced by plasma damage, leading to more cost-effective and large-scale production of Micro-LEDs. Overall, our findings contribute to the advancement of next-generation display devices and pave the way for future research in this field.

## Data Availability

Data are contained within the article.
